# Crystal structure of 4-amino­pyridinium 5-(5-chloro-2,4-dinitrophenyl)-1,3-dimethyl-2,6-dioxo-1,2,3,6-tetrahydropyrimidin-4-olate hemihydrate

**DOI:** 10.1107/S1600536814021084

**Published:** 2014-09-27

**Authors:** Manickkam Vaduganathan, Kalaivani Doraisamyraja

**Affiliations:** a PG and Research Department of Chemistry, Seethalakshmi Ramaswami College, Tiruchirappalli 620 002, Tamil Nadu, India

**Keywords:** crystal structure, barbiturate, 4-amino­pyridinium, mol­ecular salt, hydrogen bonding

## Abstract

The title mol­ecular salt, C_5_H_7_N_2_^+^·C_12_H_8_ClN_4_O_7_^−^·0.5H_2_O, crystallizes as a hemihydrate. The two rings in the barbiturate anion are not coplanar but make a dihedral angle of 43.17 (16)°. The two nitro groups deviate from the ring to which they are attached; the nitro group *ortho* with respect to the ring junction is more deviated [39.3 (4)°] than that in the *para* position [4.2 (5)°], probably to overcome steric hindrance. As a result of this, the latter nitro group is more involved in delocalizing the negative charge of the anion than the former nitro group. In the crystal, the cations and anions are linked *via* N—H⋯O hydrogen bonds forming zigzag chains along [10-1]. The chains are linked by O—H⋯O and C—H⋯O hydrogen bonds, forming slabs lying parallel to (10-1). Further C—H⋯O hydrogen bonds link the slabs, forming a three-dimensional structure.

## Abstract 2

In the title mol­ecular salt, the two rings of the barbiturate anion are inclined to one another by 43.40 (3)°. In the crystal, the cations and anions are linked *via* N—H⋯O hydrogen bonds, forming zigzag chains along [10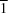], which in turn are linked by O—H⋯O and C—H⋯O hydrogen bonds, forming slabs lying parallel to (10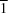).

## Chemical context   

Barbiturates occupy an important place in pharmacopoeia due to their central nervous system (CNS) depressing nature (Nogrady, 1988 ▶; Ashutoshkar, 1993 ▶; Hardman *et al.*, 2001 ▶; Yadav, 2004 ▶; Nadkarni *et al.*, 2005 ▶). Many barbiturates are ideal drugs for treating major epilepsy (Olsen *et al.*, 1986 ▶; Dhiman, 2013 ▶). In a continuation of our previous work on the synthesis of crystalline barbiturates and, in particular, similar nitro-substituted aromatic compounds (Babykala *et al.*, 2014 ▶), we report herein on the synthesis and crystal structure of the title mol­ecular salt.
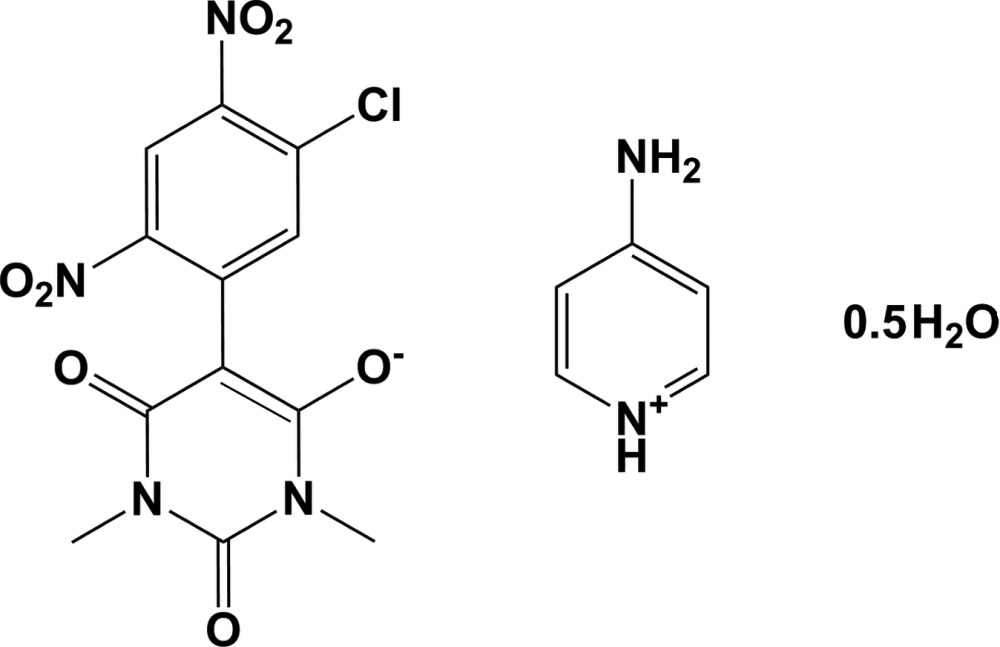


## Structural commentary   

**Figure 1:**
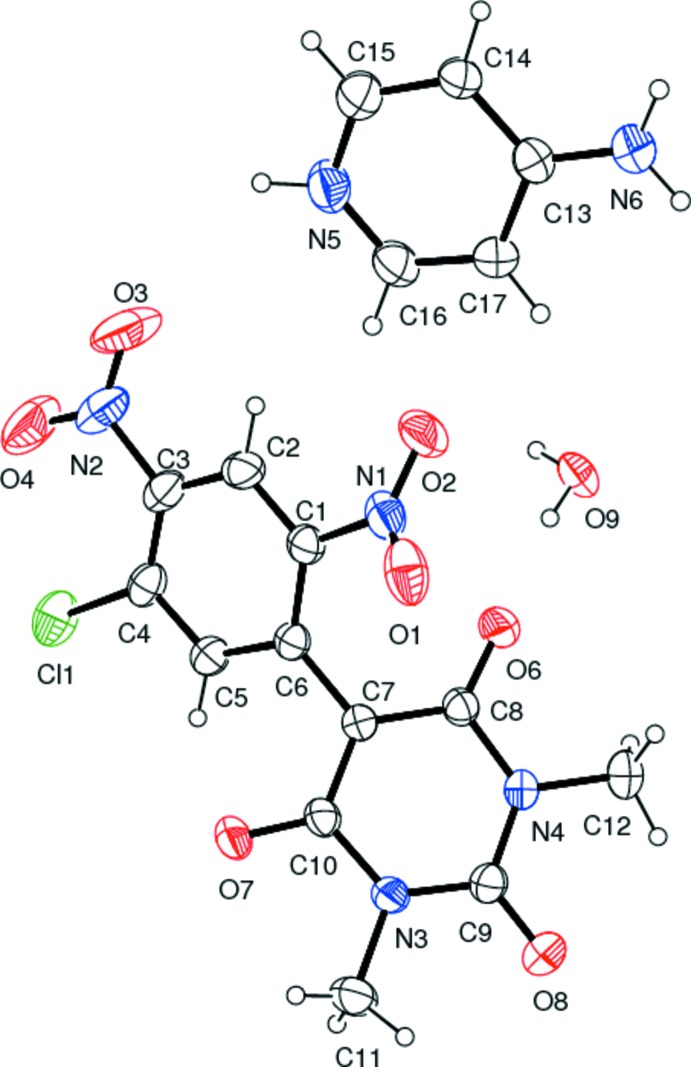
A view of the mol­ecular structure of the title salt, with atom labelling. Displacement ellipsoids are drawn at the 30% probability level.

The mol­ecular structure of the title salt is depicted in Fig. 1 ▶. The two rings in the barbiturate anion (N3/N4/C7–C10 and C1–C6) are not coplanar but are inclined to one another by 43.17 (16)°. The two nitro groups on the benzene ring (N1/O1/O2 and N2/O3/O4) deviate to different extents from the plane of the ring. The dihedral angle for the former group, adjacent to the ring junction, is 39.3 (4)° while for the later it is 4.2 (5)°. as a result of this, the latter nitro group is more involved in delocalizing the negative charge of the anion than the former. The cation is protonated at the pyridine N atom, as has been observed previously (Babykala *et al.*, 2014 ▶).

## Supra­molecular features   

**Figure 2:**
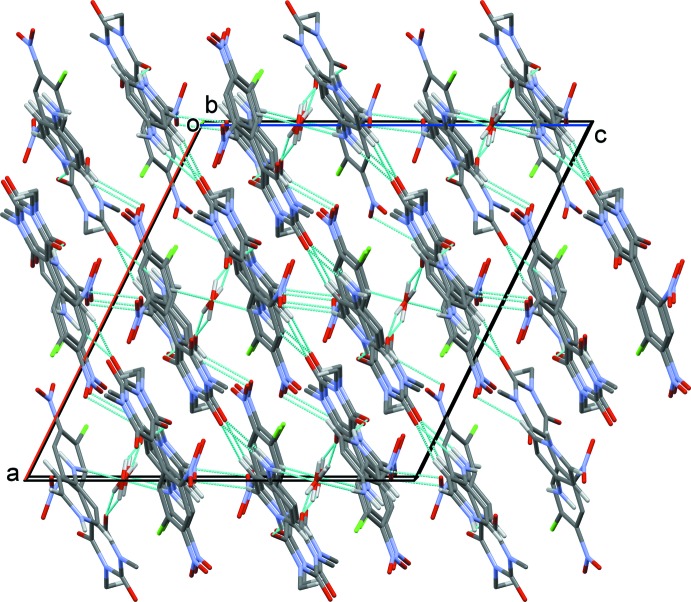
A view along the *b* axis of the crystal packing of the title compound. Hydrogen bonds are shown as dashed lines (see Table 1 ▶ for details; H atoms not involved in hydrogen bonding have been omitted for clarity).

In the crystal, the cations and anions are linked *via* N—H⋯O hydrogen bonds (Fig. 2 ▶ and Table 1 ▶) forming zigzag chains along [10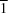]. The chains are linked by O—H⋯O and C—H⋯O hydrogen bonds, forming slabs lying parallel to (10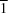). Further C—H⋯O hydrogen bonds link the slabs, forming a three-dimensional structure (Fig. 2 ▶ and Table 1 ▶).

## Database survey   

A search of the Cambridge Structural Database (Version 5.35, last update May 2014; Allen, 2002 ▶) indicated the presence of 31 hits for barbiturate salts. These including five with a substituted benzene ring in position 5 of the barbiturate. In these five compounds, the organic cations vary: tri­ethyl­ammonium with 1,3-dimethyl-2,6-dioxo-5-(2,4,6-tri­nitro­phen­yl)-1,2,3,6-tetra­hydro­pyrimidin-4-olate (LEGWIF; Rajamani & Kalaivani, 2012 ▶), 2-amino­pyridinium with 5-(5-chloro-2,4-di­nitro­phen­yl)-1,3-dimethyl-2,4-dioxo-1,2,3,4-tetra­hydropyrim­idin-6-olate (OCEWUQ; Babykala *et al.*, 2014 ▶), 2-methyl­pyridinium with 5-(2,4-di­nitro­phen­yl)-1,3-dimethyl-2,6-dioxo-1,2,3,6-tetra­hydro­pyrimidin-4-olate (YAVSOF; Sridevi & Kalaivani, 2012 ▶), *N*,*N*-di­methyl­anilinium with 1,3-dimethyl-2,6-dioxo-5-(2,4,6-tri­nitro­phen­yl)-1,2,3,6-tetrahydro­pyrimidin-4-olate (JOKGIB; Babykala *et al.*, 2014 ▶) and quinolinium with 1,3-dimethyl-2,6-dioxo-5-(2,4,6-tri­nitro­phen­yl)-1,2,3,6-tetra­hydro­pyrimidin-4-olate monohydrate (JOKGUN; Babykala *et al.*, 2014 ▶). Compound OCEWUQ is composed of the same barbiturate anion as in the title compound. The difference lies in the nature of the cation, 2-amino­pyrdinium in OCEWUQ and 4-amino­pyridinium in the title salt.

The dihedral angle between the benzene ring and the barbiturate ring varies from *ca* 42.64° in YAVSOF to *ca* 51.88° in OCEWUQ, compared to only 43.17 (16)° in the title salt. This difference is surprising considering that the barbiturate anion is the same in both OCEWUQ and the title salt.

## Synthesis and crystallization   

To 1,3-di­chloro-4,6-di­nitro­benzene (2.36 g, 0.01 mol) dissolved in 20 ml of absolute alcohol, was added 1,3-di­methyl­barbituric acid (0.01 mol, 1.56 g) dissolved in 30 ml of absolute alcohol. The mixture was heated to 313 K and 4-amino­pyridine (0.02 mol, 1.88 g) dissolved in 20 ml of absolute ethanol was added. The mixture was shaken well for 2–3 h and kept as such at 298 K. After 24 h, the excess of solvent was removed by distillation under reduced pressure and to the resulting slurry was added to 50 ml of dry ether and the mixture was refrig­erated for 5 h. The maroon-red-coloured solid obtained was filtered, powdered well and washed with 50 ml of dry ether. The dry solid was recrystallized from absolute ethanol and slow evaporation of this solvent at 293 K yielded good quality single crystals (yield 75%; m.p. 488 K).

## Refinement   

Crystal data, data collection and structure refinement details are summarized in Table 2 ▶. The cation NH and NH_2_ H atoms were located in a difference Fourier map and freely refined. The water H atoms were also located in a difference Fourier map and refined with *U*_iso_(H) = 1.2*U*_eq_(O). The C-bound H atoms were included in calculated positions and treated as riding atoms: C—H = 0.93–0.98 Å with *U*_iso_(H) = 1.5*U*_eq_(C) for methyl H atoms and = 1.2*U*_eq_(C) for other H atoms.

## Supplementary Material

Crystal structure: contains datablock(s) I, global. DOI: 10.1107/S1600536814021084/su2786sup1.cif

Structure factors: contains datablock(s) I. DOI: 10.1107/S1600536814021084/su2786Isup2.hkl

CCDC reference: 1008376

Additional supporting information:  crystallographic information; 3D view; checkCIF report

## Figures and Tables

**Table 1:** Hydrogen-bond geometry (Å, °)

*D*—H⋯*A*	*D*—H	H⋯*A*	*D*⋯*A*	*D*—H⋯*A*
N6—H6*A*⋯O8^i^	0.90 (2)	2.09 (2)	2.947 (4)	158 (4)
N6—H6*B*⋯O9^ii^	0.90 (2)	2.05 (2)	2.918 (4)	162 (4)
N5—H5*A*⋯O7^iii^	0.91 (2)	1.89 (4)	2.667 (4)	141 (5)
O9—H9*A*⋯O6^iv^	0.89 (2)	1.99 (5)	2.707 (3)	136 (6)
O9—H9*B*⋯O6	0.90 (2)	1.83 (3)	2.707 (3)	167 (9)
C15—H15⋯O4^v^	0.93	2.41	3.276 (5)	156
C16—H16⋯O2	0.93	2.46	3.190 (5)	136
C17—H17⋯O8^i^	0.93	2.56	3.290 (4)	136

Symmetry codes: (i) 
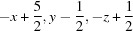
; (ii) 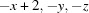; (iii) 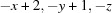; (iv) 
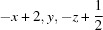
; (v) 
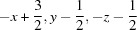
.

**Table 2:** Experimental details

Crystal data	
Chemical formula	C_5_H_7_N_2_^+^·C_12_H_8_ClN_4_O_7_^−^·0.5H_2_O
*M*_r_	459.81
Crystal system, space group	Monoclinic, *C*2/*c*
Temperature (K)	293
*a*, *b*, *c* (Å)	17.7242 (5), 14.2576 (5), 17.4321 (7)
β (°)	116.259 (3)
*V* (Å^3^)	3950.6 (2)
*Z*	8
Radiation type	Mo *K*α
μ (mm^−1^)	0.25
Crystal size (mm)	0.35 × 0.35 × 0.30

Data collection	
Diffractometer	Bruker APEXII CCD
Absorption correction	Multi-scan (*SADABS*; Bruker, 2004 ▶)
*T*_min_, *T*_max_	0.911, 0.930
No. of measured, independent and observed [*I* > 2σ(*I*)] reflections	38331, 3868, 2686
*R*_int_	0.029
(sin θ/λ)_max_ (Å^−1^)	0.617

Refinement	
*R*[*F* ^2^ > 2σ(*F* ^2^)], *wR*(*F* ^2^), *S*	0.056, 0.175, 1.07
No. of reflections	3868
No. of parameters	306
No. of restraints	7
H-atom treatment	H atoms treated by a mixture of independent and constrained refinement
Δρ_max_, Δρ_min_ (e Å^−3^)	0.47, −0.46

Computer programs: *APEX2*, *SAINT* and *XPREP* (Bruker, 2004 ▶), *SIR92* (Altomare *et al.*, 1993 ▶), *ORTEP-3 for Windows* (Farrugia, 2012 ▶), *Mercury* (Macrae *et al.*, 2008 ▶), *SHELXL97* (Sheldrick, 2008 ▶), *PLATON* (Spek, 2009 ▶) and *publCIF* (Westrip, 2010 ▶).

## supplementary crystallographic information

### Crystal data

**Table:**

C_5_H_7_N_2_^+^·C_12_H_8_ClN_4_O_7_^−^·0.5H_2_O	*F*(000) = 1896
*M**_r_* = 459.81	*D*_x_ = 1.546 Mg m^−^^3^
Monoclinic, *C*2/*c*	Mo *K*α radiation, λ = 0.71073 Å
*a* = 17.7242 (5) Å	Cell parameters from 9949 reflections
*b* = 14.2576 (5) Å	θ = 2.6–29.5°
*c* = 17.4321 (7) Å	µ = 0.25 mm^−^^1^
β = 116.259 (3)°	*T* = 293 K
*V* = 3950.6 (2) Å^3^	Block, red
*Z* = 8	0.35 × 0.35 × 0.30 mm

### Data collection

**Table:**

Bruker APEXII CCD diffractometer	3868 independent reflections
Radiation source: fine-focus sealed tube	2686 reflections with *I* > 2σ(*I*)
Graphite monochromator	*R*_int_ = 0.029
ω and φ scan	θ_max_ = 26.0°, θ_min_ = 1.9°
Absorption correction: multi-scan (*SADABS*; Bruker, 2004)	*h* = −21→21
*T*_min_ = 0.911, *T*_max_ = 0.930	*k* = −17→17
38331 measured reflections	*l* = −21→21

### Refinement

**Table:**

Refinement on *F*^2^	Secondary atom site location: difference Fourier map
Least-squares matrix: full	Hydrogen site location: inferred from neighbouring sites
*R*[*F*^2^ > 2σ(*F*^2^)] = 0.056	H atoms treated by a mixture of independent and constrained refinement
*wR*(*F*^2^) = 0.175	*w* = 1/[σ^2^(*F*_o_^2^) + (0.0628*P*)^2^ + 9.3104*P*] where *P* = (*F*_o_^2^ + 2*F*_c_^2^)/3
*S* = 1.07	(Δ/σ)_max_ < 0.001
3868 reflections	Δρ_max_ = 0.47 e Å^−^^3^
306 parameters	Δρ_min_ = −0.46 e Å^−^^3^
7 restraints	Extinction correction: *SHELXL*, Fc^*^=kFc[1+0.001xFc^2^λ^3^/sin(2θ)]^-1/4^
Primary atom site location: structure-invariant direct methods	Extinction coefficient: 0.0010 (2)

### Special details

**Table:**

Geometry. All e.s.d.'s (except the e.s.d. in the dihedral angle between two l.s. planes) are estimated using the full covariance matrix. The cell e.s.d.'s are taken into account individually in the estimation of e.s.d.'s in distances, angles and torsion angles; correlations between e.s.d.'s in cell parameters are only used when they are defined by crystal symmetry. An approximate (isotropic) treatment of cell e.s.d.'s is used for estimating e.s.d.'s involving l.s. planes.
Refinement. Refinement of *F*^2^ against ALL reflections. The weighted *R*-factor *wR* and goodness of fit *S* are based on *F*^2^, conventional *R*-factors *R* are based on *F*, with *F* set to zero for negative *F*^2^. The threshold expression of *F*^2^ > σ(*F*^2^) is used only for calculating *R*-factors(gt) *etc*. and is not relevant to the choice of reflections for refinement. *R*-factors based on *F*^2^ are statistically about twice as large as those based on *F*, and *R*-factors based on ALL data will be even larger.

### Fractional atomic coordinates and isotropic or equivalent isotropic displacement parameters (Å^2^)

**Table:**

	*x*	*y*	*z*	*U*_iso_*/*U*_eq_	Occ. (<1)
C1	0.9919 (2)	0.4245 (2)	0.09141 (19)	0.0514 (7)	
C2	0.9091 (2)	0.4328 (3)	0.0311 (2)	0.0629 (9)	
H2	0.8833	0.3839	−0.0069	0.075*	
C3	0.8647 (2)	0.5132 (3)	0.0273 (2)	0.0601 (9)	
C4	0.90337 (19)	0.5859 (3)	0.0836 (2)	0.0547 (8)	
C5	0.98582 (19)	0.5748 (2)	0.14353 (19)	0.0495 (7)	
H5	1.0111	0.6239	0.1814	0.059*	
C6	1.03389 (18)	0.4946 (2)	0.15101 (17)	0.0437 (7)	
C7	1.11882 (18)	0.48772 (19)	0.22105 (18)	0.0430 (6)	
C8	1.14416 (18)	0.4037 (2)	0.26856 (18)	0.0455 (7)	
C9	1.27389 (19)	0.4806 (2)	0.3673 (2)	0.0513 (7)	
C10	1.17194 (19)	0.5664 (2)	0.2441 (2)	0.0477 (7)	
C11	1.3064 (2)	0.6394 (3)	0.3416 (3)	0.0748 (11)	
H11A	1.2939	0.6791	0.3790	0.112*	
H11B	1.2989	0.6741	0.2915	0.112*	
H11C	1.3635	0.6180	0.3707	0.112*	
C12	1.2465 (3)	0.3194 (2)	0.3945 (2)	0.0692 (10)	
H12A	1.2993	0.3299	0.4436	0.104*	
H12B	1.2522	0.2683	0.3615	0.104*	
H12C	1.2043	0.3044	0.4128	0.104*	
C13	0.9905 (2)	0.0191 (2)	−0.1371 (2)	0.0523 (7)	
C14	0.9221 (2)	0.0263 (2)	−0.2186 (2)	0.0554 (8)	
H14	0.9062	−0.0250	−0.2554	0.067*	
C15	0.8793 (2)	0.1080 (3)	−0.2431 (2)	0.0681 (10)	
H15	0.8345	0.1122	−0.2974	0.082*	
C16	0.9629 (3)	0.1772 (2)	−0.1155 (2)	0.0678 (10)	
H16	0.9761	0.2303	−0.0811	0.081*	
C17	1.0100 (2)	0.1002 (2)	−0.0844 (2)	0.0611 (9)	
H17	1.0543	0.0998	−0.0296	0.073*	
N1	1.0356 (3)	0.3400 (2)	0.08368 (18)	0.0696 (9)	
N2	0.7784 (2)	0.5168 (4)	−0.0404 (2)	0.0866 (11)	
N3	1.24936 (15)	0.55831 (17)	0.31628 (17)	0.0516 (6)	
N4	1.22188 (16)	0.40446 (17)	0.34170 (16)	0.0501 (6)	
N5	0.8988 (2)	0.1819 (2)	−0.1926 (2)	0.0717 (9)	
N6	1.0343 (2)	−0.0582 (2)	−0.1099 (2)	0.0680 (8)	
O1	1.1078 (2)	0.34820 (19)	0.09283 (19)	0.0855 (9)	
O2	0.9948 (2)	0.26692 (19)	0.06391 (18)	0.1055 (12)	
O3	0.7499 (2)	0.4483 (3)	−0.0835 (2)	0.1351 (16)	
O4	0.7378 (2)	0.5880 (3)	−0.0505 (3)	0.1303 (15)	
Cl1	0.85552 (6)	0.68966 (8)	0.08676 (7)	0.0830 (4)	
O6	1.10297 (16)	0.33059 (16)	0.25208 (15)	0.0653 (7)	
O7	1.15599 (14)	0.64213 (15)	0.20372 (16)	0.0623 (6)	
O8	1.34014 (15)	0.47883 (18)	0.43275 (16)	0.0736 (7)	
O9	1.0000	0.1893 (2)	0.2500	0.0645 (9)	
H9A	0.948 (2)	0.211 (4)	0.235 (6)	0.077*	0.50
H9B	1.032 (3)	0.233 (4)	0.242 (5)	0.077*	0.50
H6A	1.076 (2)	−0.063 (3)	−0.0563 (14)	0.101 (16)*	
H6B	1.027 (3)	−0.108 (2)	−0.144 (2)	0.094 (14)*	
H5A	0.863 (3)	0.230 (3)	−0.219 (4)	0.15 (2)*	

### Atomic displacement parameters (Å^2^)

**Table:**

	*U*^11^	*U*^22^	*U*^33^	*U*^12^	*U*^13^	*U*^23^
C1	0.064 (2)	0.0464 (17)	0.0424 (16)	−0.0042 (14)	0.0222 (15)	0.0029 (13)
C2	0.071 (2)	0.065 (2)	0.0419 (17)	−0.0195 (18)	0.0153 (16)	0.0024 (15)
C3	0.0458 (18)	0.082 (3)	0.0455 (17)	−0.0061 (17)	0.0142 (15)	0.0130 (17)
C4	0.0445 (17)	0.071 (2)	0.0501 (17)	0.0070 (15)	0.0222 (14)	0.0110 (16)
C5	0.0481 (17)	0.0539 (17)	0.0468 (16)	0.0012 (14)	0.0213 (14)	0.0007 (14)
C6	0.0488 (16)	0.0450 (15)	0.0390 (14)	−0.0011 (12)	0.0211 (13)	0.0025 (12)
C7	0.0428 (15)	0.0395 (14)	0.0443 (15)	0.0008 (12)	0.0170 (13)	0.0007 (12)
C8	0.0475 (17)	0.0426 (16)	0.0468 (16)	−0.0002 (13)	0.0213 (14)	0.0027 (12)
C9	0.0446 (17)	0.0486 (17)	0.0564 (18)	0.0052 (13)	0.0184 (15)	−0.0006 (14)
C10	0.0465 (16)	0.0365 (15)	0.0583 (18)	0.0049 (12)	0.0214 (14)	−0.0016 (13)
C11	0.051 (2)	0.052 (2)	0.109 (3)	−0.0096 (16)	0.023 (2)	−0.006 (2)
C12	0.076 (2)	0.056 (2)	0.065 (2)	0.0086 (17)	0.0219 (19)	0.0184 (17)
C13	0.0562 (19)	0.0458 (17)	0.0588 (19)	−0.0003 (14)	0.0289 (16)	0.0030 (14)
C14	0.067 (2)	0.0455 (17)	0.0488 (17)	−0.0005 (15)	0.0206 (16)	−0.0062 (14)
C15	0.075 (2)	0.064 (2)	0.059 (2)	0.0048 (19)	0.0240 (19)	0.0018 (18)
C16	0.084 (3)	0.0486 (19)	0.067 (2)	0.0006 (17)	0.031 (2)	−0.0085 (16)
C17	0.064 (2)	0.0541 (19)	0.0539 (19)	−0.0043 (16)	0.0157 (16)	−0.0057 (15)
N1	0.114 (3)	0.0483 (17)	0.0422 (15)	0.0056 (18)	0.0302 (17)	0.0011 (12)
N2	0.056 (2)	0.130 (3)	0.057 (2)	−0.009 (2)	0.0095 (17)	0.006 (2)
N3	0.0410 (13)	0.0394 (13)	0.0662 (16)	−0.0003 (10)	0.0164 (12)	−0.0025 (12)
N4	0.0502 (15)	0.0428 (13)	0.0506 (14)	0.0046 (11)	0.0163 (12)	0.0071 (11)
N5	0.089 (2)	0.0544 (18)	0.070 (2)	0.0143 (16)	0.0329 (19)	0.0057 (15)
N6	0.072 (2)	0.0522 (17)	0.070 (2)	0.0058 (15)	0.0228 (17)	0.0007 (15)
O1	0.128 (3)	0.0666 (17)	0.085 (2)	0.0329 (18)	0.069 (2)	0.0169 (14)
O2	0.165 (3)	0.0499 (16)	0.0685 (18)	−0.0136 (18)	0.0213 (19)	−0.0118 (13)
O3	0.074 (2)	0.177 (4)	0.100 (3)	−0.016 (2)	−0.010 (2)	−0.039 (3)
O4	0.069 (2)	0.168 (4)	0.107 (3)	0.027 (2)	−0.0044 (19)	0.009 (3)
Cl1	0.0616 (6)	0.0980 (8)	0.0897 (7)	0.0296 (5)	0.0339 (5)	0.0120 (6)
O6	0.0729 (16)	0.0525 (13)	0.0598 (14)	−0.0150 (11)	0.0197 (12)	0.0101 (11)
O7	0.0590 (14)	0.0386 (12)	0.0809 (16)	0.0041 (10)	0.0232 (12)	0.0085 (11)
O8	0.0564 (15)	0.0718 (16)	0.0683 (16)	0.0030 (12)	0.0055 (13)	0.0030 (13)
O9	0.074 (2)	0.0376 (17)	0.091 (3)	0.000	0.044 (2)	0.000

### Geometric parameters (Å, º)

**Table:**

C1—C2	1.382 (5)	C12—N4	1.466 (4)
C1—C6	1.396 (4)	C12—H12A	0.9600
C1—N1	1.469 (5)	C12—H12B	0.9600
C2—C3	1.374 (5)	C12—H12C	0.9600
C2—H2	0.9300	C13—N6	1.311 (4)
C3—C4	1.384 (5)	C13—C14	1.405 (5)
C3—N2	1.463 (5)	C13—C17	1.421 (4)
C4—C5	1.379 (4)	C14—C15	1.352 (5)
C4—Cl1	1.718 (4)	C14—H14	0.9300
C5—C6	1.397 (4)	C15—N5	1.316 (5)
C5—H5	0.9300	C15—H15	0.9300
C6—C7	1.463 (4)	C16—N5	1.322 (5)
C7—C10	1.404 (4)	C16—C17	1.340 (5)
C7—C8	1.412 (4)	C16—H16	0.9300
C8—O6	1.231 (3)	C17—H17	0.9300
C8—N4	1.403 (4)	N1—O1	1.224 (4)
C9—O8	1.223 (4)	N1—O2	1.228 (4)
C9—N4	1.365 (4)	N2—O3	1.199 (5)
C9—N3	1.367 (4)	N2—O4	1.210 (5)
C10—O7	1.251 (4)	N5—H5A	0.91 (2)
C10—N3	1.397 (4)	N6—H6A	0.898 (18)
C11—N3	1.469 (4)	N6—H6B	0.896 (18)
C11—H11A	0.9600	O9—H9A	0.89 (2)
C11—H11B	0.9600	O9—H9B	0.90 (2)
C11—H11C	0.9600		
			
C2—C1—C6	122.8 (3)	H12A—C12—H12B	109.5
C2—C1—N1	115.2 (3)	N4—C12—H12C	109.5
C6—C1—N1	121.8 (3)	H12A—C12—H12C	109.5
C3—C2—C1	120.0 (3)	H12B—C12—H12C	109.5
C3—C2—H2	120.0	N6—C13—C14	122.4 (3)
C1—C2—H2	120.0	N6—C13—C17	121.0 (3)
C2—C3—C4	119.9 (3)	C14—C13—C17	116.7 (3)
C2—C3—N2	116.1 (4)	C15—C14—C13	119.6 (3)
C4—C3—N2	123.9 (4)	C15—C14—H14	120.2
C5—C4—C3	118.5 (3)	C13—C14—H14	120.2
C5—C4—Cl1	116.6 (3)	N5—C15—C14	122.2 (3)
C3—C4—Cl1	124.9 (3)	N5—C15—H15	118.9
C4—C5—C6	124.3 (3)	C14—C15—H15	118.9
C4—C5—H5	117.9	N5—C16—C17	123.6 (3)
C6—C5—H5	117.9	N5—C16—H16	118.2
C1—C6—C5	114.5 (3)	C17—C16—H16	118.2
C1—C6—C7	125.9 (3)	C16—C17—C13	118.4 (3)
C5—C6—C7	119.5 (3)	C16—C17—H17	120.8
C10—C7—C8	120.7 (3)	C13—C17—H17	120.8
C10—C7—C6	119.8 (3)	O1—N1—O2	124.7 (4)
C8—C7—C6	119.3 (3)	O1—N1—C1	118.2 (3)
O6—C8—N4	117.5 (3)	O2—N1—C1	116.9 (4)
O6—C8—C7	125.4 (3)	O3—N2—O4	122.2 (4)
N4—C8—C7	117.1 (3)	O3—N2—C3	118.5 (4)
O8—C9—N4	121.3 (3)	O4—N2—C3	119.3 (4)
O8—C9—N3	121.4 (3)	C9—N3—C10	123.8 (3)
N4—C9—N3	117.2 (3)	C9—N3—C11	117.7 (3)
O7—C10—N3	117.7 (3)	C10—N3—C11	118.4 (3)
O7—C10—C7	124.9 (3)	C9—N4—C8	123.6 (2)
N3—C10—C7	117.3 (3)	C9—N4—C12	118.8 (3)
N3—C11—H11A	109.5	C8—N4—C12	117.6 (3)
N3—C11—H11B	109.5	C15—N5—C16	119.5 (3)
H11A—C11—H11B	109.5	C15—N5—H5A	110 (4)
N3—C11—H11C	109.5	C16—N5—H5A	130 (4)
H11A—C11—H11C	109.5	C13—N6—H6A	121 (3)
H11B—C11—H11C	109.5	C13—N6—H6B	122 (2)
N4—C12—H12A	109.5	H6A—N6—H6B	116 (3)
N4—C12—H12B	109.5	H9A—O9—H9B	111 (3)
			
C6—C1—C2—C3	0.9 (5)	C13—C14—C15—N5	−0.8 (6)
N1—C1—C2—C3	−173.8 (3)	N5—C16—C17—C13	0.5 (6)
C1—C2—C3—C4	0.4 (5)	N6—C13—C17—C16	−179.8 (4)
C1—C2—C3—N2	178.1 (3)	C14—C13—C17—C16	−0.5 (5)
C2—C3—C4—C5	−1.0 (5)	C2—C1—N1—O1	137.5 (3)
N2—C3—C4—C5	−178.5 (3)	C6—C1—N1—O1	−37.2 (4)
C2—C3—C4—Cl1	−179.4 (3)	C2—C1—N1—O2	−38.7 (4)
N2—C3—C4—Cl1	3.1 (5)	C6—C1—N1—O2	146.5 (3)
C3—C4—C5—C6	0.5 (5)	C2—C3—N2—O3	5.4 (5)
Cl1—C4—C5—C6	179.1 (2)	C4—C3—N2—O3	−177.0 (4)
C2—C1—C6—C5	−1.3 (4)	C2—C3—N2—O4	−175.4 (4)
N1—C1—C6—C5	173.0 (3)	C4—C3—N2—O4	2.2 (6)
C2—C1—C6—C7	174.5 (3)	O8—C9—N3—C10	−175.5 (3)
N1—C1—C6—C7	−11.2 (5)	N4—C9—N3—C10	5.3 (5)
C4—C5—C6—C1	0.6 (4)	O8—C9—N3—C11	1.4 (5)
C4—C5—C6—C7	−175.5 (3)	N4—C9—N3—C11	−177.8 (3)
C1—C6—C7—C10	143.2 (3)	O7—C10—N3—C9	178.3 (3)
C5—C6—C7—C10	−41.2 (4)	C7—C10—N3—C9	−3.4 (4)
C1—C6—C7—C8	−41.3 (4)	O7—C10—N3—C11	1.4 (4)
C5—C6—C7—C8	134.4 (3)	C7—C10—N3—C11	179.7 (3)
C10—C7—C8—O6	−178.1 (3)	O8—C9—N4—C8	178.1 (3)
C6—C7—C8—O6	6.4 (5)	N3—C9—N4—C8	−2.7 (4)
C10—C7—C8—N4	3.5 (4)	O8—C9—N4—C12	0.2 (5)
C6—C7—C8—N4	−172.0 (2)	N3—C9—N4—C12	179.4 (3)
C8—C7—C10—O7	177.0 (3)	O6—C8—N4—C9	179.9 (3)
C6—C7—C10—O7	−7.5 (5)	C7—C8—N4—C9	−1.6 (4)
C8—C7—C10—N3	−1.2 (4)	O6—C8—N4—C12	−2.2 (4)
C6—C7—C10—N3	174.3 (3)	C7—C8—N4—C12	176.4 (3)
N6—C13—C14—C15	180.0 (3)	C14—C15—N5—C16	0.8 (6)
C17—C13—C14—C15	0.7 (5)	C17—C16—N5—C15	−0.6 (6)

### Hydrogen-bond geometry (Å, º)

**Table:**

*D*—H···*A*	*D*—H	H···*A*	*D*···*A*	*D*—H···*A*
N6—H6*A*···O8^i^	0.90 (2)	2.09 (2)	2.947 (4)	158 (4)
N6—H6*B*···O9^ii^	0.90 (2)	2.05 (2)	2.918 (4)	162 (4)
N5—H5*A*···O7^iii^	0.91 (2)	1.89 (4)	2.667 (4)	141 (5)
O9—H9*A*···O6^iv^	0.89 (2)	1.99 (5)	2.707 (3)	136 (6)
O9—H9*B*···O6	0.90 (2)	1.83 (3)	2.707 (3)	167 (9)
C15—H15···O4^v^	0.93	2.41	3.276 (5)	156
C16—H16···O2	0.93	2.46	3.190 (5)	136
C17—H17···O8^i^	0.93	2.56	3.290 (4)	136

Symmetry codes: (i) −*x*+5/2, *y*−1/2, −*z*+1/2; (ii) −*x*+2, −*y*, −*z*; (iii) −*x*+2, −*y*+1, −*z*; (iv) −*x*+2, *y*, −*z*+1/2; (v) −*x*+3/2, *y*−1/2, −*z*−1/2.
